# Neck Circumference as an Anthropometric Indicator of Central Obesity in Patients with Prediabetes: A Cross-Sectional Study

**DOI:** 10.1155/2019/4808541

**Published:** 2019-06-09

**Authors:** Thunyarat Anothaisintawee, Nakarin Sansanayudh, Sangsulee Thamakaison, Dumrongrat Lertrattananon, Ammarin Thakkinstian

**Affiliations:** ^1^Department of Family Medicine, Faculty of Medicine, Ramathibodi Hospital, Mahidol University, Bangkok, Thailand; ^2^Cardiology Unit, Department of Internal Medicine, Phramongkutklao Hospital, Bangkok, Thailand; ^3^Section for Clinical Epidemiology and Biostatistics, Faculty of Medicine, Ramathibodi Hospital, Mahidol University, Bangkok, Thailand

## Abstract

Measurement of waist circumference has substantial variability and some limitations, while neck circumference is a simple and reliable anthropometric measure. This study aimed to assess the association between neck circumference and waist circumference and to identify the best cutoff of neck circumference that could predict central obesity in prediabetic patients. This cross-sectional study included adult patients with prediabetes, defined as having fasting plasma glucose levels ranging from 100 to 125 mg/dL or HbA1c ranging from 5.7 to 6.49%, who visited the outpatient clinic of Family Medicine Department, Ramathibodi Hospital, Thailand, during October 2014 and March 2016. Neck circumference was measured from the level just below the laryngeal prominence perpendicular to the long axis of the neck. Central obesity was defined as having waist circumference measurements greater than 90 and 80 cm for males and females, respectively. The correlation between neck circumference and waist circumference was explored by applying pairwise correlation coefficient. Receiver operating characteristic (ROC) curve analysis was performed and Youden index equal to “sensitivity – (1-specificity)” was calculated. Neck circumference that yielded the maximum Youden index was determined as the optimal cutoff point for prediction of central obesity. There were 1,534 patients eligible for this study. After adjusting for covariables, neck circumference was found to be significantly associated with waist circumference in both females and males, with *β*-coefficients of 1.01 (95% CI: 0.83, 1.20) and 0.65 (95% CI: 0.46, 0.85), respectively. After applying the ROC analysis, neck circumferences ≥ 32 cm in females and ≥ 38 cm in males were determined as the best cutoff values to predict central obesity. Neck circumference is strongly correlated with waist circumference in prediabetics and should be considered as an alternative to the waist circumference measurement in screening for central obesity.

## 1. Introduction

Prediabetes is an intermediate dysglycemic state between normal glucose regulation and overt type 2 diabetes. Prediabetes is very common in the general population, with its prevalence ranging from approximately 25% to 45% [1]. Patients with prediabetes have high risk of converting to diabetes [2, 3], as the annual risk of prediabetes developing diabetes is > 4 times greater than the risk of those with normal glucose tolerance developing diabetes [4]. Furthermore, subjects with prediabetes also have greater risk of having cardiovascular events. In a meta-analysis, prediabetes significantly increases the risk of cardiovascular disease, with the pooled increase in the relative risk of approximately 20% [5].

The main pathophysiology underlying prediabetes is insulin resistance [6]. Central obesity, which is characterized by excessive accumulation of abdominal adipose tissue, is significantly associated with insulin resistance [7] and is also a strong risk factor of type 2 diabetes mellitus and cardiovascular diseases [8]. Previous evidence showed that waist circumference (WC) is strongly correlated with the amounts of abdominal fat. Waist circumference has been used as the standard method to define central obesity, according to the NCEP ATP III guideline [9]. However, measurement of WC has substantial variability and certain limitations. For instance, there is no consensus regarding the standard method of WC and, thus, different techniques and locations of measurement could result in varying values of WC [10]. Therefore, there is a need for a better alternative parameter that can be used as a screening tool for central obesity and insulin resistance.

Neck circumference (NC) is a simple, reliable, and widely available anthropometric measure. Neck circumference has been shown in previous studies to be associated with central obesity in the general population [11–16] and also with visceral adipose tissue (VAT) measured by CT scan [17]. VAT is closely related with insulin resistance and the risk of cardiovascular diseases. Furthermore, in a study of severely obese subjects, NC surpassed other anthropometric measurements, including WC, as a powerful marker of both VAT and insulin resistance [18]. Recently, NC has gained substantial interest and has been studied in many subgroups of patients. There has been, however, no study of the relationship between NC, WC, and central obesity in prediabetic patients before.

The objective of this study is to assess the correlation between NC and central obesity and to identify the best cutoff NC value associated with central obesity in prediabetic patients.

## 2. Methods

### 2.1. Study Setting and Participants

This cross-sectional study used the baseline data of a prediabetes cohort study that was carried out at the outpatient clinic of Family Medicine Department, Ramathibodi Hospital, Bangkok, Thailand, during October 2014 and March 2016. Patients with prediabetes, defined as having fasting plasma glucose (FPG) levels ranging from 100 to 125 mg/dL or HbA1c ranging from 5.7 to 6.49% [19], were included in this study. Patients were excluded if their waist and neck circumferences could not be measured, if they were diagnosed with malignancy or thyroid diseases, or if they were not willing to participate in the study. All patients signed the written informed consent. The study's protocol was approved by the ethics committee of Faculty of Medicine, Ramathibodi Hospital, Mahidol University.

### 2.2. Data Collection

Baseline characteristics of this study's participants (i.e., age, sex, marital status, educational level, history of smoking, and alcohol drinking) were collected by interviewing them with trained staffs. Medical records were reviewed by trained physicians (TA, DL, and ST) to determine the past medical history (i.e., hypertension, dyslipidemia, chronic kidney disease (CKD), coronary artery disease (CAD), and cerebrovascular disease (CVA)) of this study's participants.

Weight and height were measured without shoes to the nearest 100 g and 0.1 cm, respectively. Body mass index (BMI) was calculated by dividing weight in kilograms with the square of height in meters. Association between BMI and body fat percentage differs between Asian and European populations, as at BMI lower than 25 kg/m^2^. Asian populations have a greater risk of developing type 2 diabetes and cardiovascular diseases than European populations. Therefore, the Asian-Pacific BMI cutoff points between 23 and 25 kg/m2 and greater than 25 kg/m2 were applied to classify overweight and obesity in this study [20, 21]. Systolic and diastolic blood pressures were measured with an automatic blood pressure monitored by trained nurses after the participants had rested for at least 15 minutes. Waist circumference (cm) was measured from the middle point between the lowest rib and iliac crest in standing position. Neck circumference (cm) was measured using nonstretchable plastic tape to the nearest 1 mm and was measured from the level just below the laryngeal prominence perpendicular to the long axis of the neck with head positioned in Frankfurt horizontal plane. Central obesity was defined as WC measurements greater than 90 cm and 80 cm for males and females, respectively [22].

### 2.3. Laboratory Assessment

The most recent laboratory values (i.e., FPG, triglyceride, high-density lipoprotein cholesterol (HDL-C), and low-density lipoprotein cholesterol (LDL-C)) were retrieved from laboratory databases, Medical Statistic Unit, Ramathibodi Hospital. Fasting plasma glucose and triglyceride levels were measured using hexokinase glucose-6 phosphate dehydrogenase and lipase/glycerol kinase glycerol-3 phosphate oxidase methods, respectively. HDL-C and LDL-C levels were measured using the accelerator selective detergent method.

### 2.4. Statistical Analysis

Baseline characteristics of this study's participants were presented as frequencies and percentages for categorical variables, while continuous variables with normal distributions were presented as means and standard deviations (SD). Correlation between NC and WC was examined using the pairwise correlation coefficient. Associations between NC and other covariables (i.e., age, BMI, FPG, triglyceride, HDL-C, and LDL-C levels) and WC were explored by applying linear regression model. Variables that had P-values of less than 0.1 were considered in multivariate linear regression to explore the independent relationship between those variables and WC.

Receiver operating characteristic (ROC) curve analysis was performed and Youden's index that was equal to “sensitivity – (1-specificity)” was calculated. Neck circumference that yielded the maximum Youden's index was determined as the optimal cutoff point for prediction of central obesity. Multivariate logistic regression was applied to explore the independent relationship between the NC that was above the cutoff point and central obesity. Possible confounding factors (i.e., age, BMI, FPG, triglyceride, HDL-C, and LDL-C levels) were considered in the multivariate logistic regression model, if they had the P-value less than 0.10 from univariate model. Odds ratio (OR) was estimated by the exponential *β*-coefficient from the logistic regression analysis. Performances of NC and BMI for the prediction of central obesity were compared using the ROC analysis.

All statistical analyses were stratified by sex (i.e., male and female) except for logistic regression model. Two-sided test with P-value less than 0.05 was determined as the level of significance. All statistical tests were performed using STATA program version 15.

## 3. Results

In total, 1,534 patients with prediabetes were recruited from the outpatient clinic of Family Medicine Department, Ramathibodi Hospital, during October 2014 and March 2016. Thirty-one patients were excluded because their NC and WC could not be measured. Additionally, 87 patients who were diagnosed with thyroid diseases and 28 patients who were diagnosed with cancer were excluded from the study (see Supplementary Figure 1). Characteristics of this study's participants are presented in Table 1. Mean age and BMI of the participants were 62.32 years (SD = 8.75) and 25.96 kg/m^2^ (SD = 4.02), respectively. Mean WC (93.22 cm; SD = 9.52) and NC (38.35 cm; SD = 3.26) in males were higher than mean WC (88.12 cm; SD = 10.06) and NC (33.97 cm; SD = 2.82) in females. Nearly all participants (91%) were diagnosed with dyslipidemia, defined as serum TG ≥ 150 mg/dL and/or LDL-C ≥ 160 mg/dL and/or total cholesterol ≥ 200 mg/dL and/or patients taking lipid lowering drugs. Approximately 67% of study's participants had hypertension, while only 4%, 0.37%, and 1% of participants had been diagnosed with CKD, CAD, and CVA, respectively. Mean FBS and LDL-C levels were 105.94 mg/dL (SD = 7.93) and 127.03 mg/dL (SD = 31.76), which were not significantly different between males and females, while mean HDL-C level was higher in females (56.77 mg/dL; SD = 14.03) than in males (49.33 mg/dL; SD = 12.64).

### 3.1. Association between Neck Circumference and Waist Circumference

Neck circumference was shown to be significantly correlated with WC, with correlation coefficient of 0.62 (P-value<0.001) (see Supplementary Figure 2). Beta-coefficients of NC and other factors from univariate linear regression are illustrated in Supplementary Table 1. NC was positively associated with WC in both females and males (*β*-coefficient=2.19, P-value<0.001 in females and *β*-coefficient=1.82, P-value<0.001 in males). All factors (i.e., age, BMI, SBP, DBP, FPG, TG, HDL-C, and LDL-C), except age in females, had the P-values less than 0.10 and were considered in multivariate linear regression. After adjusting for confounding factors, NC remained significantly associated with WC in both females and males. *β*-coefficients were 1.01 (95% CI: 0.83, 1.20) in females and 0.65 (95% CI: 0.46, 0.85) in males, suggesting that every 1 cm increase of NC increased WC by approximately 1 cm in females and 0.65 cm in males.

BMI (*β*-coefficient = 1.33; 95% CI: 1.21, 1.45) and TG level (*β*-coefficient = 0.01; 95% CI: 0.004, 0.02) were also significantly associated with WC in female from multivariate analysis, while age (*β*-coefficient = 0.07; 95% CI: 0.02, 0.12), DBP (0.08; 95% CI: 0.02, 0.13), and BMI (*β*-coefficient = 1.75; 95% CI: 1.57, 1.93) were significantly associated with WC in males (see Table 2).

### 3.2. Neck Circumference and Central Obesity

**Figure 1:**
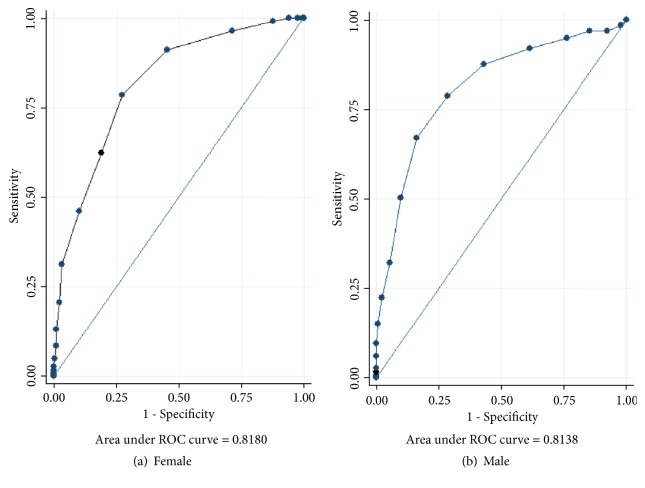
Receiver operating characteristic curve of neck circumference for prediction of central obesity.

By applying the ROC analysis, NC ≥ 32 cm in females and ≥ 38 cm in males were determined as the best cutoff values to predict central obesity. Areas under ROC curve (AUC) for females and males were 0.82 (95% CI: 0.79, 0.85) and 0.81 (95% CI: 0.78, 0.85), respectively (see Figures 1(a) and 1(b)). Sensitivities of NC ≥ 32 cm in females and ≥ 38 cm in males for predicting central obesity were 0.79 and 0.67, while specificities were 0.73 and 0.84, respectively. Positive predictive value (PPV), negative predictive value (NPV), likelihood ratio positive (LR+), likelihood ratio negative (LR-), and Youden's index of NC ≥ 32 cm in females were 89%, 61.20%, 2.93, 0.29, and 0.51, respectively. PPV, NPV, LR+, LR-, and Youden's index of NC ≥ 38 cm in males were 84%, 35.12%, 4.19, 0.39, and 0.51, respectively.

Results from univariate logistic regression showed that NC, BMI, SBP, DBP, triglyceride, HDL-C, and FPG were significantly associated with central obesity and were considered in multivariate logistic regression analysis (see Supplementary Table 2). After adjusting for other covariables (i.e., BMI, SBP, DBP, triglyceride, HDL-C, and FBS level), NC ≥32 cm in females and ≥38 cm in males remained significantly associated with central obesity, with odds ratio of 6.83 (95% CI: 5.01, 9.31). In addition, BMI ≥ 23 kg/m2, DBP, and HDL-C were also found to be significantly associated with central obesity from multivariate regression analysis with ORs of 6.40 (95% CI: 4.65, 8.83), 1.02 (95% CI: 1.003, 1.04), and 0.98 (95% CI: 0.97, 0.99), respectively (see Table 3).

When compared with BMI, AUC of NC (0.75; 95% CI: 0.73-0.78) for predicting central obesity was higher than AUC of BMI (0.74; 95% CI: 0.72-0.77). However, this difference was not statistically significant.

## 4. Discussion

Our results found that NC was independently associated with WC in prediabetes patients. NC ≥ 32 cm in females and ≥ 38 cm in males were determined as the best cutoff values to predict central obesity. These cutoff values had moderate accuracy for diagnosis of central obesity with AUC of 0.82 (95% CI: 0.79, 0.85) for females and 0.81 (95% CI: 0.78, 0.85) for males. In addition, NC ≥32 cm in females and ≥38 cm in males were significantly associated with central obesity in prediabetic patients with odds ratio of 6.83 (95% CI: 5.01, 9.31).

Waist circumference is a well-accepted measure for the screening of central obesity. Waist circumference is currently recommended by all guidelines to be used as the main criteria for the diagnosis of metabolic syndrome. However, at present, there is no consensus regarding the standard technique and location of measurement for WC. Waist circumference is affected substantially by measurement technique and locations of WC measurement [23–25]. Position, meals, and respiration could all affect the measurement of WC [26]. Furthermore, WC also could not be used with pregnant individuals or those with ascites. In patients who are bed-ridden, measurement of WC could be more difficult and the supine position also has an impact on the circumference of the patient's abdomen. Since there has been no research to identify the cutoff or technique of WC in supine position, WC could be greatly different when measured in supine versus upright position and, thus, WC limits its use in many patients who are unable to stand upright. Moreover, WC requires removal of the cloth which may be inconvenient in some situations. Therefore, there is a continuing search for a novel and simple anthropometric measurement that could overcome these limitations of WC and could be used as better practical screening tools for central obesity and insulin resistance in clinical practice.

Neck circumference is an emerging measure that has received substantial interest recently. Unlike WC, which has vast diversity in its location of measurement, the location of NC measurement is standard and straightforward. Meal, respiration, and position have no effect on NC measurement. NC could be measured without requirement for cloth removal and could also be measured in pregnant and ascitic patients. The minor limitation of neck circumference is that it could not be used on patients with neck mass (e.g., goiter or cervical lymphadenopathy) or with thyroid diseases.

The previous studies in the general population have consistently shown the association between NC and WC [11–16]. There were only few studies regarding NC and WC in diabetic patients. Yang et al. showed association between NC and WC in 3,182 Chinese diabetic patients [27]. Furthermore, Aswathappa et al. found that NC in diabetics is significantly different from nondiabetics [15]. Neck circumference was shown to be associated with WC in both diabetics and nondiabetics but the cutoff for diabetics was different and was higher than the cutoff for nondiabetics. However, there has been no previous study of NC and WC or central obesity in population of prediabetes.

There were many strong reasons for studying the relationship between NC and WC in this important subgroup of prediabetics patients. First, prediabetes has very high prevalence and affects substantial number of individuals in general population [1]. Second, this dysglycemic condition poses a risk factor of type 2 diabetes [2–4]. Third, prediabetes per se also has greater risk of cardiovascular events compared to normoglycemic people [5]. Fourth, the main pathophysiology underlying prediabetes is insulin resistance [6] which could be simply screened for by using WC measurement. WC is currently recommended as a screening tool for insulin resistance and is used for diagnosis of the metabolic syndrome. However, measurement of WC has many limitations, as described previously. Therefore, there is a need for alternative measurement method that is simple, cheap and widely available and that does not have the limitations that WC measurement has. Fifth, although NC has received increased attention as an alternative to WC and has good preliminary results in many studied populations, there has been no previous study comparing NC and WC in patients with prediabetes before.

In this study, we conducted a cross-sectional study in 1,534 prediabetic patients. The mean age of prediabetics in this study was around 60 years and approximately two-thirds were female. The mean BMI was around 25. Two-thirds of the study's population had hypertension and most of them had dyslipidemia. Most patients did not have history of vascular diseases.

This study found that NC strongly correlates with WC, even after the results were adjusted for other parameters including BMI. Similarly, previous studies from many ethnic populations found that there was a significant association between NC and WC in the general population [11–16] and studies conducted in diabetic patients also found that there is a strong correlation between NC and WC [15, 27]. The results from this study of prediabetic patients are consistent with those of previous studies and, thus, help fill the gap of knowledge in this subgroup of patients.

Regarding the cutoffs of NC to be used in clinical practice, we found that NC ≥ 32 cm in females and ≥ 38 cm in males were the best cutoffs for identifying central obesity, with adjusted OR of 6.83. Previous studies reported different cutoffs of NC, which are possibly due to the difference in their studied populations. From SABPA study, Hoebel et al. found that the appropriate cutoffs for predicting the metabolic syndrome in young versus old subjects and in different ethnic groups were different [28]. Therefore, using different cutoffs according to the studied population might be the best approach. The large observational studies, which are needed in order to determine the optimal cutoff for NC, are lacking and there was no meta-analysis of the NC cutoff at the time of writing this manuscript. However, after reviewing the available publications, it should be noted that most of the previous studies suggested the cutoffs of 33-35 cm in females and 37-39 cm in males [11, 12, 27], which were similar to the cutoffs found in our study. There was only one previous study of NC in the Thai population, which was conducted in a university setting of 587 subjects who attended a healthy aging clinic in the northeastern province of Thailand [29]. They found that the best cutoff values of NC that were associated with the metabolic syndrome were 33 cm in females and 39 cm in males, which are in agreement with our cutoffs. The results of this study are consistent with those of previous studies and, thus, suggest the potential role of using this study's cutoffs for NC measurement in screening of central obesity.

There were some strengths of this study. First, it is the first study of NC and WC in prediabetic patients. Second, the sample size was adequate, which allowed for adjusting for other factors in multivariate analysis. Third, the details regarding other factors that might influence the WC and central obesity were meticulously collected and these parameters were used in multivariate analysis.

This study also had few limitations. First, there was no data regarding plasma insulin and insulin resistance status in the study population. Previous studies consistently reported the association between NC and HOMA-IR [11–13, 16, 30–33]. Neck circumference was found to be more significantly correlated with HOMA-IR than with WC [32]. Patients with prediabetes generally have higher level of insulin resistance compared to general population and it would be interesting to see the relationship between insulin resistance and NC in prediabetes.

Second, there was no data regarding visceral adipose tissue, which is known to be strongly correlated with insulin resistance. Previous studies reported significant association between NC and visceral adipose tissue [17, 33, 34]. The information of visceral adipose tissue and NC in prediabetics would be beneficial for further understanding the underlying pathophysiology and the relation between NC and metabolic abnormality in prediabetics.

Third, this study was conducted in a single center. We encourage more research with larger numbers of prediabetic subjects to confirm the findings found in our study. Lastly, due to the cross-sectional design of this study, the prognostic value of NC for predicting the risk of conversion to diabetes could not be assessed. Cho et al. found that high baseline NC increased the risk of newly diagnosed diabetes in a 10-year follow-up of nondiabetic subjects [16]. Additionally, in Framingham cohort, Preis et al. followed 2,732 subjects for 10 years and found that large NC significantly increased risk of developing newly diagnosed diabetes [33]. Neck circumference has also been reported to be associated with future cardiovascular events. Dai et al. found that high NC increased risk of cardiovascular events and mortality in 12,151 high-risk cardiology outpatients who were followed up for 8.8 years [35].

Prediabetics have a high conversion rate to become diabetics. It would be interesting to see the clinical benefit of using NC as a predictor of diabetes conversion in prediabetes. Currently, we are conducting a prospective follow-up study of this group of prediabetics and are systematically collecting the incidence of diabetes as well as cardiovascular outcomes. We hope that this simple measurement of NC measurement might play an important role in screening prediabetic patients at risk and leads to early intervention to prevent the conversion to diabetes and its complications.

## 5. Conclusion

Neck circumference was found to be strongly correlated with WC in prediabetic patients, even after the results were adjusted for other parameters including BMI. Neck circumference ≥ 32 cm in female and that ≥ 38 cm in male were the best cutoffs for identifying patients with central obesity. Neck circumference could be considered as an alternative to WC measurement in screening of central obesity.

## Figures and Tables

**Table 1:** Description of characteristics of study's participants.

Factor	Female (N = 1,007)	Male (N= 527)	Total (N = 1,534)
Age; year (mean; SD)	62.41 (8.04)	62.15 (9.98)	62.32 (8.75)

Alcohol drinking (%)			
Current	122 (12)	206 (39)	328 (21)
Past	178 (18)	223 (42)	401 (26)
Never	706 (70)	98 (19)	804 (53)

Smoking (%)			
Current	13 (1)	57 (11)	70 (5)
Past	39 (4)	264 (50)	303 (20)
Never	955 (95)	206 (39)	1161 (75)

*Underlying diseases*			
Hypertension (%)	667 (67)	361 (69)	1,028 (67)
Dyslipidemia (%)	916 (91)	473 (90)	1389 (91)
CKD (%)	18 (2)	42 (8)	60 (4)

*Physical examination (mean; SD)*			
BMI (kg/m^2^)	26.17 (4.25)	25.63 (3.55)	25.96 (4.02)
WC (cm)	88.12 (10.06)	93.22 (9.52)	89.87 (10.17)
NC (cm)	33.97 (2.82)	38.35 (3.26)	35.47 (3.64)
SBP (mmHg)	132.71 (17.11)	133.47 (15.27)	132.97 (16.50)
DBP (mmHg)	78.44 (8.69)	80.16 (10.15)	79.03 (9.25)

*Laboratory (mean; SD)*			
FPG (mg/dL)	105.75 (8.03)	106.29 (7.73)	105.94 (7.93)
TG (mg/dL)^a^	123 (22-444)	127 (36-880)	124 (22-880)

HDL-C (mg/dL)	56.77 (14.03)	49.33 (12.64)	54.26 (14.02)
LDL-C (mg/dL)	128.22 (32.26)	124.73 (30.46)	127.03 (31.76)

^a^Median (range).

BMI, body mass index; CKD, chronic kidney disease; DBP, diastolic blood pressure; FPG, fasting plasma glucose; HDL-C, high-density lipoprotein cholesterol; LDL-C, low-density lipoprotein cholesterol; NC, neck circumference; SBP, systolic blood pressure; SD, standard deviation; TG, triglyceride; WC, waist circumference.

**Table 2:** Multivariate linear regression analysis between waist circumference and other factors.

Factors	Female		Male	
	B-coefficient (95% CI)	P-value	B-coefficient (95% CI)	P-value
Age	NA^a^	NA^a^	0.07 (0.02, 0.12)	0.011
DBP	-0.02 (-0.07, 0.03)	0.493	0.08 (0.02, 0.13)	0.004
NC	1.01 (0.83, 1.20)	<0.001	0.65 (0.46, 0.85)	<0.001
BMI	1.33 (1.21, 1.45)	<0.001	1.75 (1.57, 1.93)	<0.001
Triglyceride	0.01 (0.004, 0.02)	0.002	0.002 (-0.004, 0.01)	0.483

BMI, body mass index; DBP, diastolic blood pressure; NC, neck circumference.

^a^Age was not included in multivariate linear regression analysis for female due to its P-value >0.1 from univariate analysis.

**Table 3:** Multivariate logistic regression between central obesity and other factors.

Factors	Odds ratio (95% CI)			P-value
	Female	Male	Total	
DBP	1.02 (1.002-1.05)	1.02 (0.99-1.05)	1.02 (1.003-1.04)	0.020
NC^a^	5.67 (3.73-8.63)	5.43 (3.43-8.59)	6.83 (5.01-9.31)	<0.001
BMI^b^	7.34 (4.92-10.94)	6.02 (3.51-10.34)	6.40 (4.65-8.83)	<0.001
HDL-C	0.97 (0.96-0.98)	0.99 (0.97-1.004)	0.98 (0.97-0.99)	0.002

^a^Neck circumference <32 versus ≥32 cm in females and <38 versus ≥38 cm in males.

^b^BMI <24 versus ≥24 kg/m^2^.

BMI, body mass index; CI, confidence interval; DBP, diastolic blood pressure; HDL-C, high-density lipoprotein cholesterol.

## Data Availability

The data used to support the findings of this study have not been made available because this study is a part of ongoing cohort study.

## Supplementary Materials

Supplementary Materials
* Supplementary Figure 1.* Flow chart of study participants.* Supplementary Figure 2.* Scatter plot of neck circumference and waist circumference.* Supplementary Table 1.* Univariate linear regression analysis between waist circumference and factors.* Supplementary Table 2.* Univariate logistic regression between central obesity and factors.
