# Supplementary material for: Neck Circumference as an Anthropometric Indicator of Central Obesity in Patients with Prediabetes: A Cross-Sectional Study
Source: Biomed Res Int. 2019 Jun 9;2019:4808541. doi: 10.1155/2019/4808541 (PMC6590547; doi:10.1155/2019/4808541)
Supplement: Supplementary Materials — Supplementary Figure 1. Flow chart of study participants. Supplementary Figure 2. Scatter plot of neck circumference and waist circumference. Supplementary Table 1. Univariate linear regression analysis between waist circumference and factors. Supplementary Table 2. Univariate logistic regression between central obesity and factors. [file 4808541.f1.docx]

**Supplementary Figure 1.** Flow chart of study participants

**Supplementary Figure 2.** Scatter plot of neck circumference and waist circumference

**Supplementary Table 1.** Univariate linear regression analysis between waist circumference and factors

| **Factors** | **Female** | | **Male** | |
| --- | --- | --- | --- | --- |
|  | **B-coefficient (95% CI)** | **P-value** | **B-coefficient (95% CI)** | **P-value** |
| Age | -0.02 (-0.10, -0.05) | 0.554 | -0.11 (-0.19, -0.03) | 0.009 |
| SBP | 0.05 (0.02, 0.09) | 0.005 | 0.10 (0.05, 0.15) | <0.001 |
| DBP | 0.14 (0.07, 0.21) | <0.001 | 0.20 (0.12, 0.28) | <0.001 |
| NC | 2.19 (2.02, 2.37) | <0.001 | 1.82 (1.63, 2.02) | <0.001 |
| BMI | 1.74 (1.64, 1.84) | <0.001 | 2.11 (1.97, 2.25) | <0.001 |
| Triglyceride | 0.03 (0.02, 0.04) | <0.001 | 0.01 (0.001, 0.02) | 0.026 |
| LDL-C | -0.02 (-0.04, 0.002) | 0.073 | -0.02 (-0.05, 0.003) | 0.081 |
| HDL-C | -0.16 (-0.20, -0.12) | <0.001 | -0.17 (-0.24, -0.10) | <0.001 |
| FPG | 0.20 (0.13, 0.28) | <0.001 | 0.13 (0.02, 0.23) | 0.018 |

BMI, body mass index; CI, confidence interval; DBP, diastolic blood pressure; FPG, fasting plasma glucose; HDL-C, high-density lipoprotein cholesterol; LDL-C, low-density lipoprotein cholesterol; NC, neck circumference; SBP, systolic blood pressure

**Supplementary Table 2.** Univariate logistic regression between central obesity and factors

| **Factors** | **Odds ratio** | **95% CI** | **P-value** |
| --- | --- | --- | --- |
| Age | 0.99 | 0.98-1.00 | 0.123 |
| SBP | 1.012 | 1.004-1.019 | 0.002 |
| DBP | 1.03 | 1.02-1.05 | <0.001 |
| NC^*^ | 11.73 | 8.96, 15.37 | <0.001 |
| BMI** | 12.70 | 9.61-16.80 | <0.001 |
| Triglyceride | 1.005 | 1.003-1.007 | <0.001 |
| LDL-C | 1.00 | 0.996-1.003 | 0.683 |
| HDL-C | 0.973 | 0.965-0.981 | <0.001 |
| FPG | 1.03 | 1.02-1.05 | <0.001 |

*Neck circumference <32 vs ≥32 cm in females and <38 vs ≥38 cm in males

**BMI <23 vs ≥23 kg/m^2^

BMI, body mass index; CI, confidence interval; DBP, diastolic blood pressure; FPG, fasting plasma glucose; HDL-C, high-density lipoprotein cholesterol; LDL-C, low-density lipoprotein cholesterol; NC, neck circumference; SBP, systolic blood pressure
